# Roles played by IL-8 in altering dynamics of trabecular meshwork cells after human cytomegalovirus infection

**DOI:** 10.3389/fcimb.2025.1550509

**Published:** 2025-04-25

**Authors:** Fumie Ehara, Daisuke Nagase, Masachika Kai, Kaori Adachi, Hitomi Miyake, Yumiko Shimizu, Yoshitsugu Inoue, Dai Miyazaki

**Affiliations:** ^1^ Ophthalmology and Visual Science, Tottori University Faculty of Medicine, Yonago, Japan; ^2^ Research Initiative Center, Organization for Research Initiative and Promotion, Tottori University Faculty of Medicine, Yonago, Japan

**Keywords:** cytomegalovirus, glaucoma, trabecular meshwork, CXCL8, CCL2, Cdc42, IL-8

## Abstract

Open-angle glaucoma (OAG) is the leading cause of blindness worldwide. Human cytomegalovirus (HCMV) is known to infect the trabecular meshwork cells (HTMCs) and corneal endothelial cells leading to chronic and recurrent elevations of the intraocular pressure (IOP) as secondary glaucoma. To investigate how HCMV affects the function of HTMCs, we analyzed the effects of HCMV infection on cultured HTMCs infected with the endothelial-adapted strain, TB40/E, of HCMV. We studied the induced molecular mechanisms focusing on the OAG-associated chemokines, IL-8 and CCL2. The HTMCs were analyzed for transcriptome changes using RNAseq analysis. Our results showed that HCMV infection activated interferon signaling and significantly increased the expression of IL-8 and CCL2. The IL-8-responsive transcriptional pathway was analyzed by using a CXCR2 antagonist which is associated with cellular movement and development of the hematological system. In contrast, the CCL2-sensitive pathway, assessed using a CCR2 antagonist, was linked to olfactory receptor signaling and keratinization. HCMV infection activated cell motility with the formation of lamellipodia and filopodia. The infection-induced activation of cell motility was dependent on both CXCR2 and CCR2, and IL-8 stimulated filopodia-mediated cell motility. HCMV infection also induced cell contraction that was dependent on CXCR2, but not on CCR2, and it involved the activation of Rac1/Cdc42. These results suggest that HCMV infection altered the cytoskeletal dynamics and contraction of the HTMCs in a CCR2- and CXCR2-dependent manner. These changes have the potential of causing an increase in the resistance to aqueous humor outflow in HCMV-associated anterior uveitis and corneal endotheliitis.

## Introduction

Human cytomegalovirus (HCMV) is a relatively common herpesvirus that is latent in a large proportion of the general population. Immunocompromised patients are especially susceptible to HCMV infections, and it is increasingly recognized that HCMV can cause serious ocular complications even in immunocompetent individuals. Among these complications are HCMV-induced corneal endotheliitis, anterior uveitis, and secondary glaucoma (Ye et al., 2023)

Corneal endotheliitis is characterized by corneal edema, keratic precipitates, and elevated intraocular pressure (IOP), and it can progress to endothelial cell loss and corneal decompensation (Koizumi et al., 2015). HCMV-induced corneal endotheliitis is manifested as recurrent episodes of intraocular inflammation leading to a chronic and potential blindness if left untreated. The underlying pathogenesis of endotheliitis is a viral infection of the corneal endothelial cells that triggers an immune response and subsequent endothelial dysfunction (Miyazaki et al., 2017)

HCMV anterior uveitis is another significant manifestation of a HCMV infection. This condition typically presents as a recurrent, unilateral anterior uveitis along with anterior chamber inflammation and increased IOP. This condition may be manifested as a Posner-Schlossman-like syndrome. The pathogenesis of the anterior uveitis involves direct infection of the trabecular meshwork cells (TMCs) leading to an increase in the resistance to outflow from the anterior chamber resulting in an increase in the IOP. In addition, the recurrent inflammation causes fibrotic changes of the TMCs enhancing the resistance to outflow that result in an elevation of the IOP.

Screening for biomarkers of open angle glaucoma (OAG) has been performed on the aqueous humor of the eyes of patients with OAG. A number of studies have concluded that there was a significant association of OAG with inflammatory cytokines and chemokines (Chua et al., 2012; Ghanem et al., 2011; Kuchtey et al., 2010; Sawada et al., 2010; Chono et al., 2018). Of these, interleukin-8 (IL-8) and CCL2 have been consistently reported to be associated with OAG and intraocular pressure (IOP) elevations. Importantly, HCMV infection of the TMCs has been shown to upregulate IL-8 and CCL2 which then alters the cytoskeletal dynamics and increases the outflow resistance resulting in an elevation of the IOP (Li et al., 2012; Chono et al., 2018; Lee et al., 2021). These findings corroborated the idea that these chemokines can be used as diagnostic markers of OAG with or without an infection.

Given the significant impact of HCMV on the anterior segment of the eye, there is a critical need to determine the molecular mechanisms underlying HCMV-induced endotheliitis and secondary glaucoma especially in immunocompetent adults.

Thus, the purpose of this study was to investigate the roles IL-8 and CCL2 played in modulating the dynamics of the TMCs. To accomplish this, we infected cultured HTMCs with the TB40/E strain of HCMV and examined the biochemical pathways that are associated with an increase in the outflow resistance and IOP elevation.

## Results

**Figure 1:**
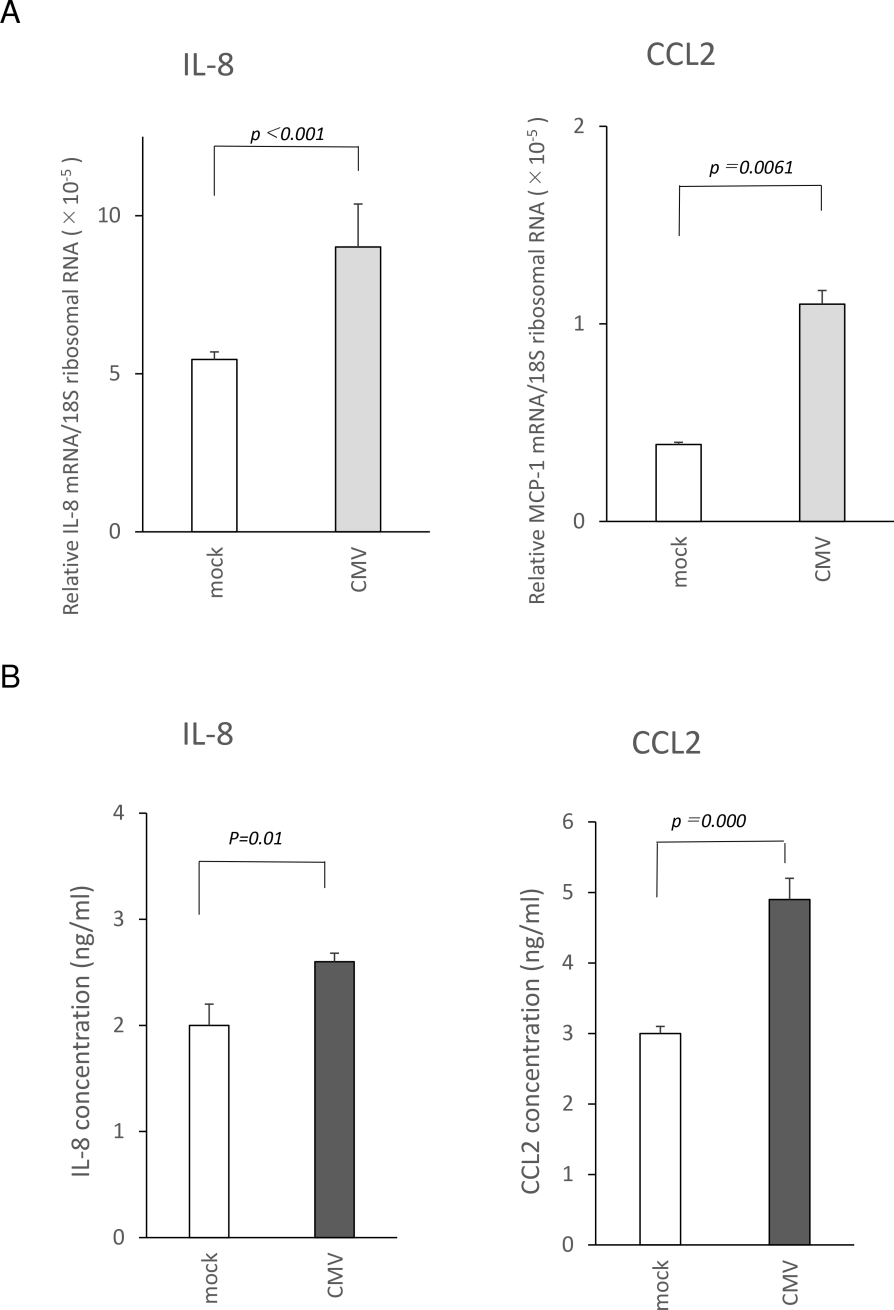
Induction of IL-8 and CCL2 in human trabecular meshwork cells (HTMCs) after human cytomegalies virus (HCMV) infection. HTMCs (2×10^5^ cells/well) were infected with TB40/E strain, and the supernatant was collected at 12 h post infection, and the cells were extracted for mRNA. The induction of the mRNA of IL-8 and CCL2 was determined by real-time RT-PCR **(A)**. N = 8/group. Secretion of IL-8 and CCL2 was determined using ELISA **(B)**. N = 6/group. The infection of HTMCs increased the m-RNA expression of IL-8 and CCL2, and stimulated secretion of IL-8 and CCL2, significantly. t-test. Data are representative of repeated experiments.

We first determined whether the mRNAs of human trabecular meshwork cells (HTMCs) were able to induce the expression of IL-8 and CCL2 using RT-PCR (**Figure 1A**). Our results indicated that both IL-8 and CCL2 mRNA were significantly upregulated at 12 h post infection (pi; **Figure 1**). To confirm that the proteins of IL-8 and CCL2 were induced, the supernatants of the infected HTMCs were assayed by ELISA (**Figure 1B**). Our results indicated that the IL-8 levels were significantly elevated at 12 h pi (HCMV, 2.6 ± 0.08 ng/ml; mock, 2.0 ± 0.2 ng/ml, *P* = 0.01). The level of CCL2 was also significantly elevated (HCMV, 4.9 ± 0.3 ng/ml; mock, 3.0 ± 0.1 ng/ml, *P* = 0.000).

Next, we examined the molecular responses of HTMCs induced by HCMV infection. For this, the RNA of HCMV-infected HTMCs were analyzed for comprehensive transcriptional responses using RNAseq analysis. After the HCMV infection, 367 genes were induced or repressed (fold change >1.5, false discovery rate (FDR) <0.05) compared to mock infection after 12 hours. Of these, 65 genes were significantly upregulated, and the highest was *OASL* followed by *HERC5* and *IFIT2*. The highest downregulated gene was *USP53* followed by *ARMH1* and *NADK2*.

**Figure 2:**
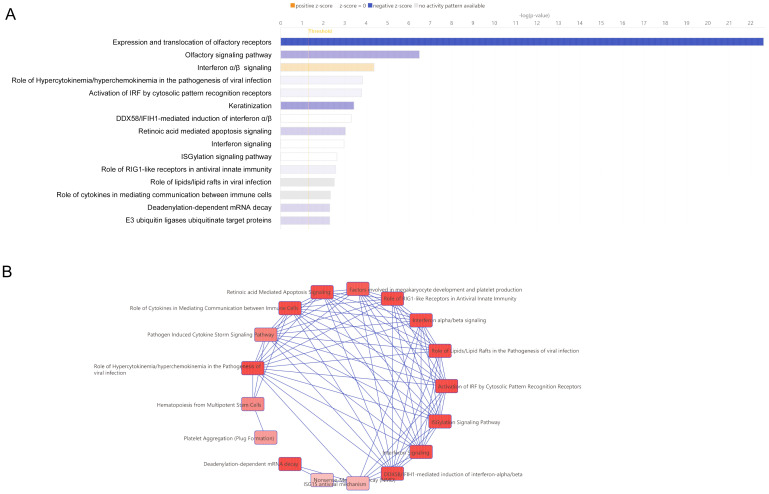
Canonical analysis of human trabecular meshwork cells (HTMCs) after HCMV infection by RNA-seq analysis. **(A)** The significantly infected genes (fold change >1.5, FDR<0.05) underwent Ingenuity pathway analysis. Association with each canonical pathway was assessed by the z-score. The top15 significant canonical pathways are shown. Canonical pathway analysis indicated significant positive association was present for interferon α/β signaling by the z-score (orange bar). Canonical pathway, including the expression and translocation of olfactory receptors, olfactory signaling pathway, role of hypercytokinemia/hyperchemokinemia in the pathogenesis of viral infection, activation of IRF by cytosolic pattern recognition receptor, and keratinization were negatively associated. **(B)** All of the significant canonical pathways were analyzed for associations as networks. The largest network of canonical pathways is shown as overlapping pathways. This network of canonical pathways is characterized by interferon α/β signaling, role of hypercytokinemia/hyperchemokinemia in the pathogenesis of viral infection, and activation of IRF by cytosolic pattern recognition receptor.

When canonical pathway analysis was conducted for HCMV infection-induced genes, the interferon α/β signaling pathway was activated (orange bar, **Figure 2A**). In contrast, the canonical networks including the expression and translocation of the olfactory receptors, olfactory signaling pathway, role of hypercytokinemeia/hyperchemokinemia in the pathogenesis of viral infection, and activation of IRF by cytosolic pattern recognition receptors were repressed (blue bar, **Figure 2A**). The other canonical networks are summarized as anti-viral immune responses of the acquired and innate arms.

To determine how all significant canonical pathways were associated with the other pathways, the overlapping canonical pathways are shown in **Figure 2B**. The largest clusters of canonical pathways are also shown in **Figure 2B**. In these networks, Interferon α/β signaling was highly associated with the role of hypercytokinemeia/hyperchemokinemia in the pathogenesis of viral infections, and the activation of Interferon regulatory factors (IRF) by cytosolic pattern recognition receptors (**Figure 2B**). These findings indicated that there was a strong association of chemokines with the anti-viral immune responses.

IL-8 and CCL2 are well known chemokines reported as markers for OAG. (Chua et al., 2012, Ghanem et al., 2011, Kuchtey et al., 2010, Sawada et al., 2010, Chono et al., 2018) Therefore, we next examined the roles played by IL-8 in HCMV-infected HTMCs. The cells were assessed by blocking IL-8 by an inhibitor of CXCR2 which is a major receptor of IL-8 on non-neutrophil lineage cells. Application of SB225002, a potent and selective CXCR2 antagonist, to HCMV-infected HTMCs resulted in a differential expression of 296 genes compared to CMV-infected HTMCs; 157 downregulated and 139 upregulated (FDR <0.05). The highest repressed genes were *TRPA1, PPP3CB, CRNKL1, ARL5A, and CEP83*. The data used in this study can be accessed at the following link: https://zenodo.org/records/14543758.

**Figure 3:**
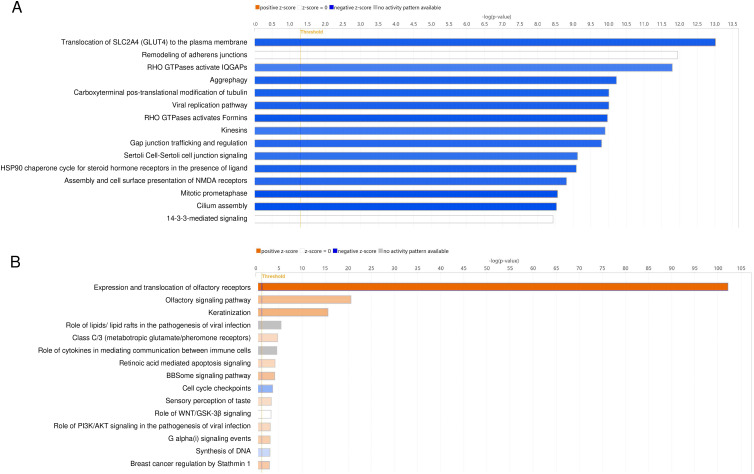
Network analysis of the roles of CXCR2 and CCR2 in human trabecular meshwork cells (HTMCs) after HCMV infection. HCMV-infected HTMCs blocked by CXCR2 or CCR2 were analyzed using RNA-seq analysis. The significantly induced or repressed genes were assessed for canonical analysis using Ingenuity pathway analysis. Associations with detected canonical pathways were assessed by the z-scores. **(A)** The top repressed canonical pathways by CXCR2 blockade by SB225002 were the translocation of SLC2A4 (GLUT4) to the plasma membrane, remodeling of adherens junctions, Rho GTPases activated IQGAPs, aggrephagy, carboxyterminal post-translational modification of tubulin, viral replication pathway, Rho GTPases activates Formins, kinesins, and Gap junction trafficking and regulations (blue bars). **(B)** The repressed canonical pathways by CCR2 blockade by RS504393 were the cell cycle checkpoints and synthesis of DNA.

Canonical network analysis was performed to determine how blocking CXCR2 affected the canonical pathways. The highly repressed canonical networks were the translocation of SLC2A4 (GLUT4) to the plasma membrane, RHO GTPases activation of IQGAPs, aggrephagy, and carboxyterminal post-translational modification of tubulin (blue bar, **Figure 3A**). Other repressed pathways were related to cell motility and trafficking. Remodeling the adherens junctions were observed for both of repressed and stimulatory effects (white bar, **Figure 3A**). Collectively, the inhibition of CXCR2 was significantly associated with a remodeling of the extracellular matrix, cell motility, and glucose uptake for energy consumption.

To determine the roles played by CCL2, we examined the effects of inhibiting CCR2 on HCMV-infected HTMCs. Exposure of HTMCs to RS504393, a CCR2 antagonist, resulted in the differential expression of 839 genes compared to CMV-infected HTMCs; 112 downregulated and 727 upregulated (FDR <0.05). The highest repressed genes were *MMP3, CENPS/CENPS-CORT, E2F8, CDCA4*, and *SHC3*.

We then assessed the canonical networks associated with the CCR2 blockade. Our results showed that CCR2 blockade resulted in an activation of canonical networks including the expression and translocation of olfactory receptors and olfactory signaling pathways (orange bar, **Figure 3B**). These pathways are known to have immune modulatory role for macrophages and monocytes that are known to express CCR2 (Orecchioni et al., 2022)

CCR2 blockade was also associated with the canonical pathways, the roles of lipids/lipid rafts in the pathogenesis of viral infections, and the roles of cytokines in mediating communication between immune cells were associated. However, no activity pattern was observed (gray bar, **Figure 3B**). The pathways repressed by CCR2 blockade were cell cycle checkpoints and synthesis of DNA (blue bar, **Figure 3B**). Collectively, blocking CXCR2 was highly associated with the cell motility associated pathways. However, the pathways related to cell motility were not significantly observed after blocking CCR2.

**Figure 4:**
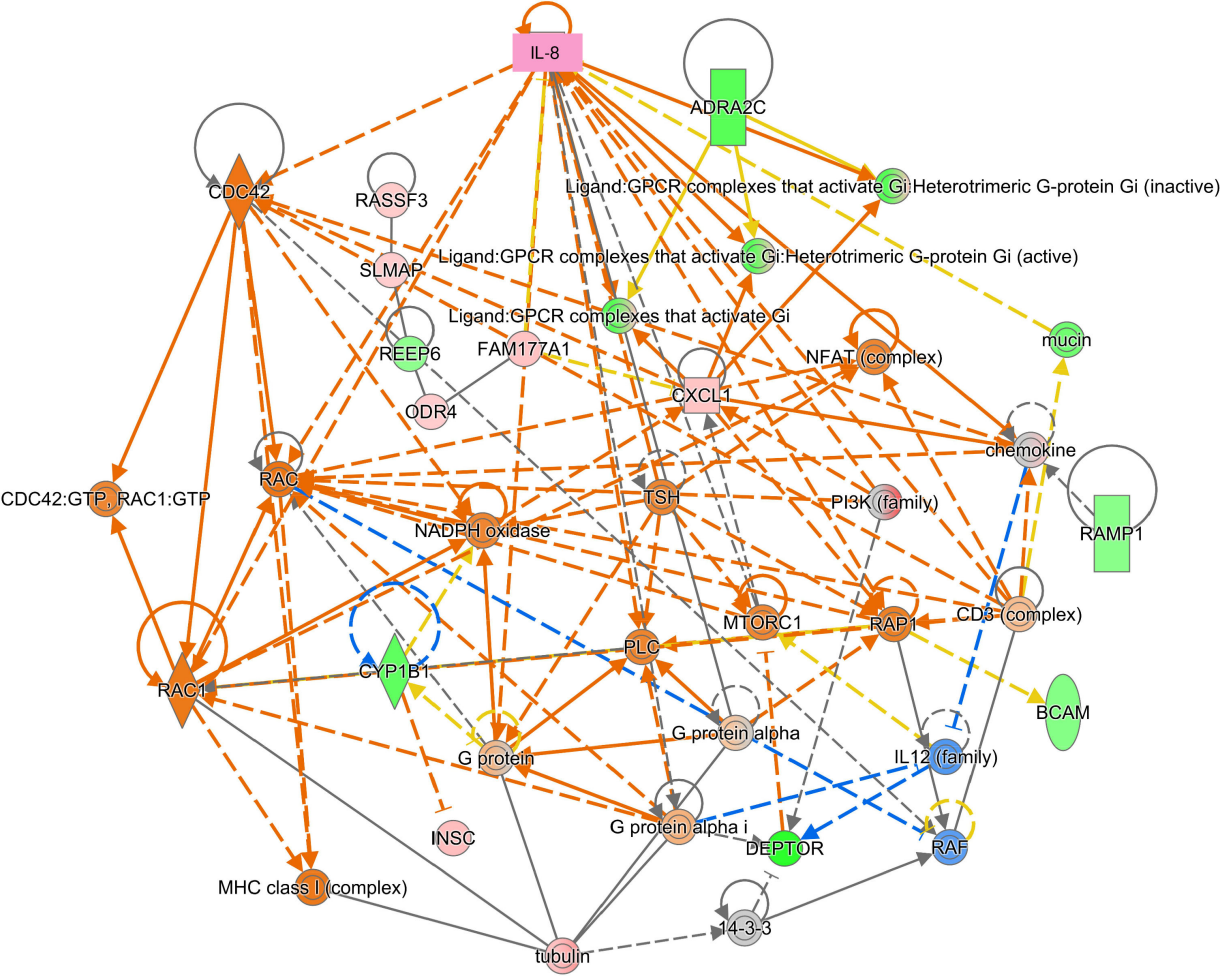
Network analysis of repressed genes after CXCR2 blockade in human trabecular meshwork cells (HTMCs) after HCMV infection. The significantly induced or repressed genes after CXCR2 blockade were assessed for network analysis. IL-8 associated network was characterized as cell trafficking and cell-to-cell signaling and interaction. IL-8 (CXCL8) was shown associated with Cdc42 and Rac.

We then conducted network analyses using genes that were differentially expressed after the receptor blockade. To determine the roles of CXCR2 in HCMV-infected HTMCs, the differentially expressed genes after SB225002 blockade were analyzed by network analysis. Our results showed that IL-8/CXCR2 was significantly associated with the network of cell trafficking, cell-to-cell signaling and interaction, and hematological system development and function (*P* = 10^-20^, **Figure 4**). In this network, IL-8 (CXCL8) was found to be associated with CDC42 and RAC as downstream signaling sites (**Figure 4**). When network analyses using differentially expressed genes after CCR2 blockade was determined, associated networks were not appreciably observed for CCL2/CCR2.

**Figure 5:**
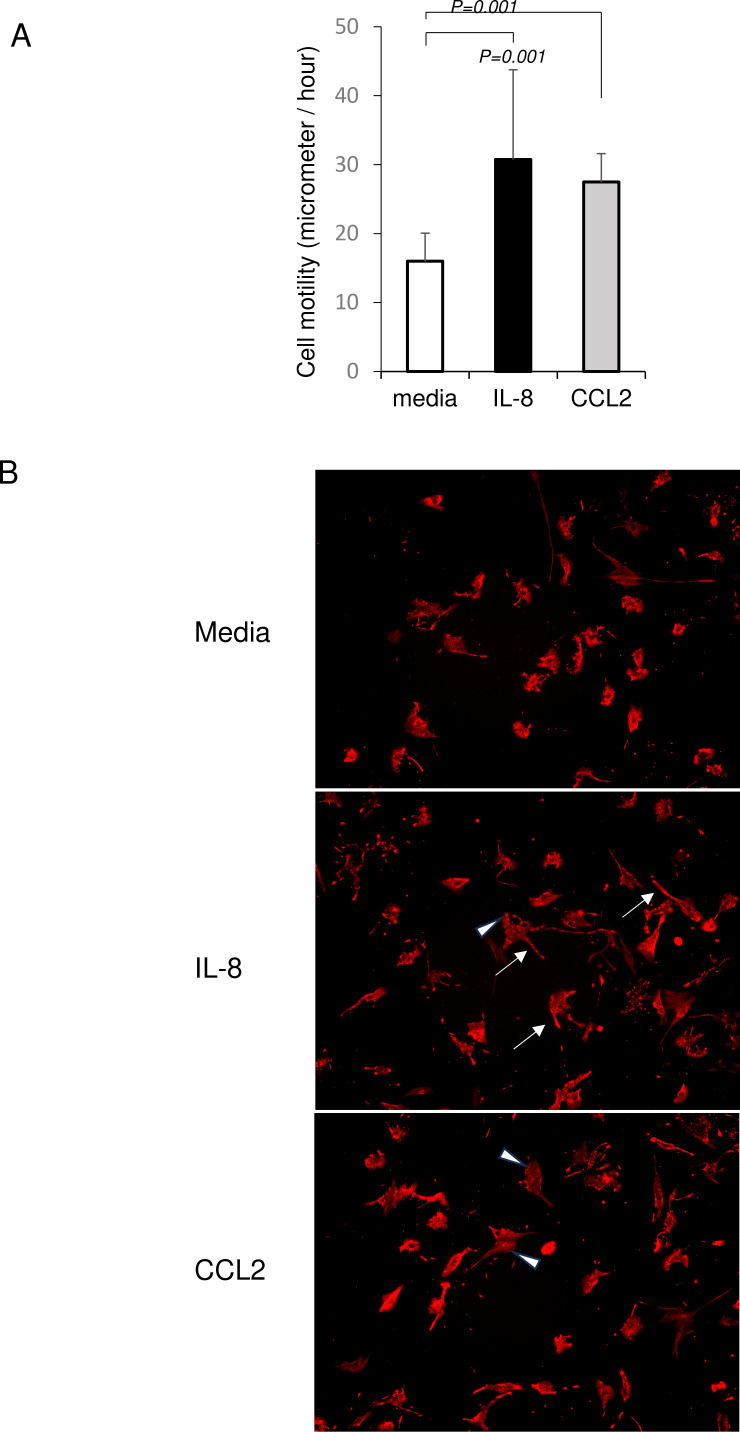
Chemokine-induced cell motility of human trabecular meshwork cells (HTMCs). HTMCs were stained for actin using SiR-actin. **(A)** Exposure to IL-8 (10 ng/ml) and CCL2 (10 ng/ml) significantly stimulated the speed of cell movements (micrometers/hour). The data and images represent the speed of cell movement at 6 hours post-treatment. For analysis, 4 three-dimensional frames of 1410 μm × 1060 μm × 90 μm were randomly selected from the wells by a masked observer, and cells whose movement could be traced within the same frame over 24 hours were extracted for digitization. Next, a randomly selected eight representative cells per group were analyzed by a masked observer for their speed of cell movement at each hourly interval using a multivariate linear regression analysis. P-values were adjusted using the Scheffe test. Data represent repeated experiments. Representative cell motility was shown in supplementary video files. **(B)** IL-8-stimulated HTMC were accompanied by an increased formation of lamellipodia (arrowhead) and filopodia (arrow). CCL2- stimulated HTMC were mainly accompanied by formation of lamellipodia (arrowhead).

To determine the roles of IL-8 and CCL2, HTMCs were examined for their responses to their exposure to these cytokines. We first determined the effect of IL-8 or CCL2 on cell motility. To do this, HTMCs were stained for actin, and time lapse imaging was conducted after exposure to IL-8 or CCL2. Our results showed that both IL-8 and CCL2 stimulated cell movement and the formation of stress fibers (**Figure 5**, supplementary video). The IL-8-induced increased motility was accompanied by an increased formation of lamellipodia and filopodia (**Figure 5B**). These are broad flat protrusions from the cells and thin actin rich protrusions. In contrast, CCL2 induced an increase in the motility with lamellipodia formation.

**Figure 6:**
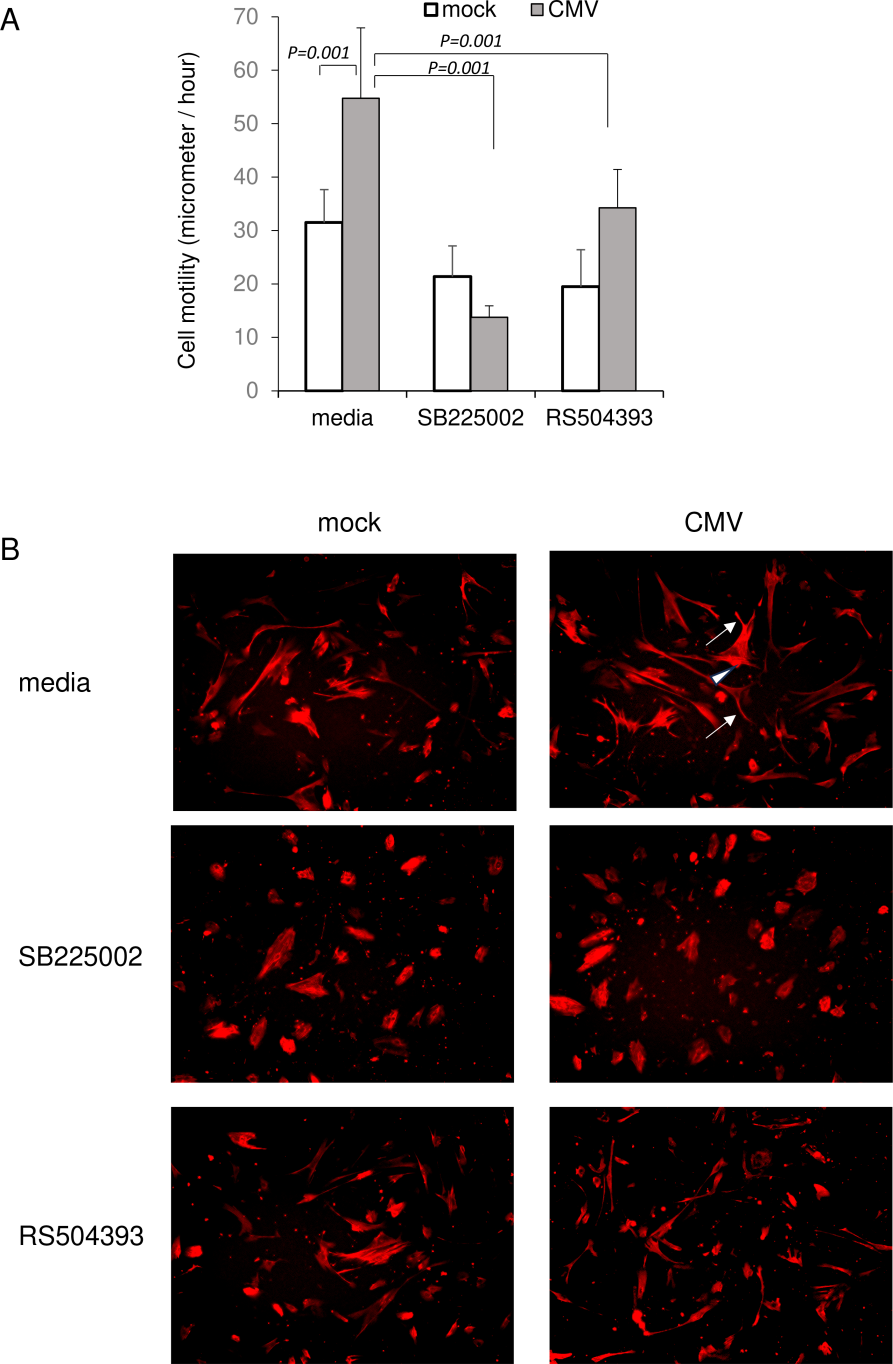
HCMV-induced cell motility of human trabecular meshwork cells (HTMCs). HTMCs infected with TB40/E were stained for actin using SiR-actin. **(A)** HCMV infected HTMCs were assessed for cell motility. HCMV infection significantly stimulated speed of cell movement. Exposure to SB225002 (500 nM) abolished HCMV-induced cell motility. The blockade of CCR2 by RS504393 (5 μM) partially reduced cell motility by HCMV infection. The data and images represent the speed of cell movement at 6 hours post-infection. For analysis, 4 three-dimensional frames of 1410 μm × 1060 μm × 90 μm were randomly selected from the wells by a masked observer, and cells whose movement could be traced within the same frame over 24 hours were extracted for digitization. Next, a randomly selected eight representative cells per group were analyzed by a masked observer for their speed of cell movement at each hourly interval using a multivariate linear regression analysis. P-values were adjusted using the Scheffe test. Data represent repeated experiments. **(B)** HCMV-infected HTMC were accompanied by an increased formation of lamellipodia (arrowhead) and filopodia (arrow). Exposure to SB225002 (500 nM) suppressed formation of lamellipodia and filopodia.

We then examined whether HCMV infection affected the cell motility in the same way. Our results showed that HCMV infection enhanced the cell motility significantly with the formation of lamellipodia and filopodia (**Figure 6A**, **Supplementary video**). This increased cell motility was blocked by SB225002, an antagonist to CXCR2. A blockade of CCR2 by RS504393 partially, but significantly, reduced cell motility. Blocked by SB225002 also suppressed infection-induced formation of lamellipodia and filopodia (**Figure 6B**). These findings indicated that the HCMV-induced activation of cell movement was mediated by CXCR2 or CCR2.

**Figure 7:**
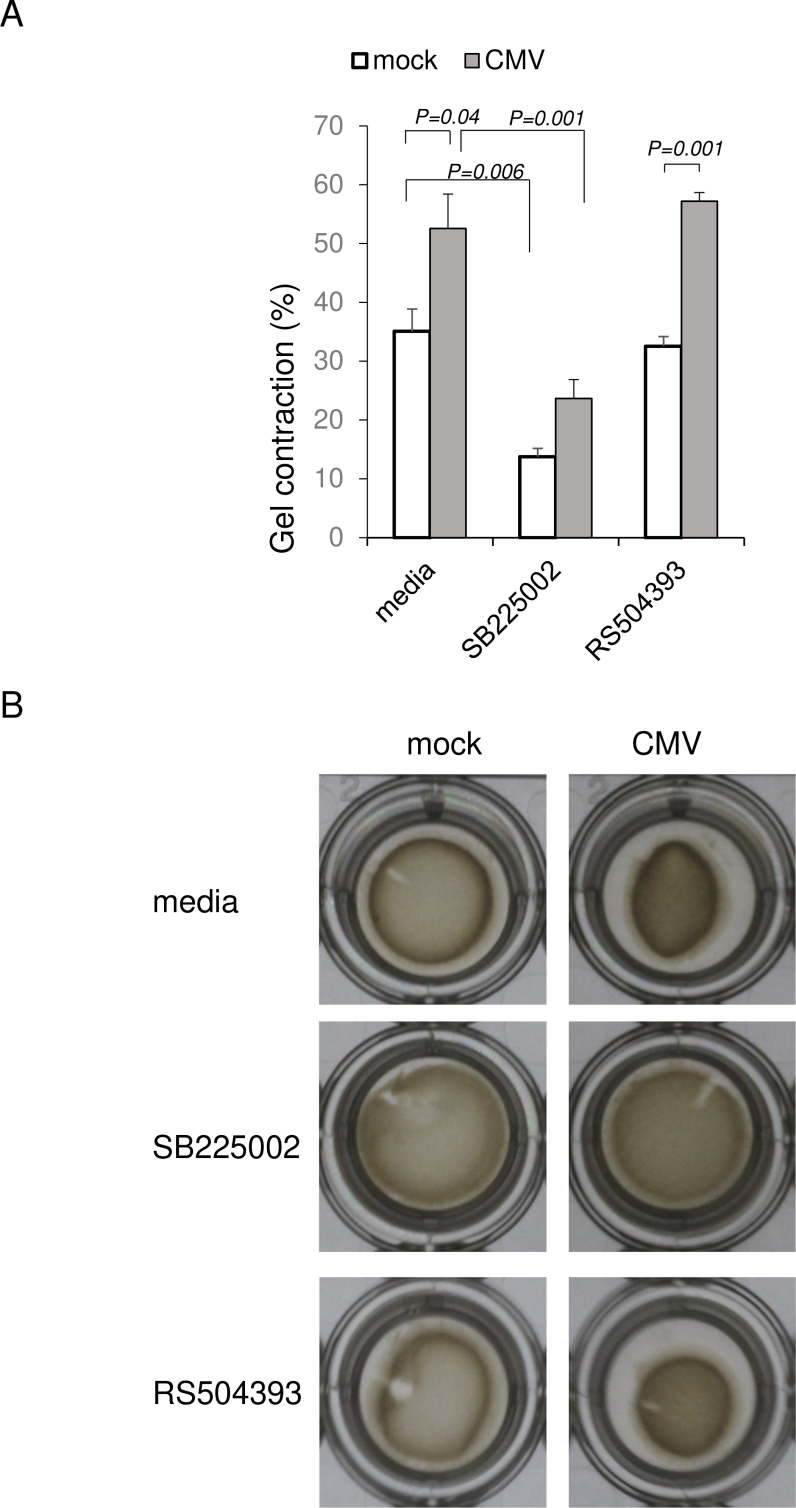
HCMV induced contraction of trabecular meshwork cells. HCMV-infected HTMCs were embedded in collagen gel, and assessed for cell contraction at 6 h. Cell contraction was calculated by the percentage of collagen gel area subtracted from total area of the well. HCMV (TB40/E) infection significantly induced cell contraction. Cell contraction was suppressed by blocking CXCR2 by SB225002 (500 nM). In contrast, cell contraction was not affected by RS504393 exposure. N=6/group. ANOVA and Tukey test. Data represent repeated experiments.

The intraocular pressure (IOP) is associated with the TMCs, and a contraction of the cells increases the resistance to outflow. Therefore, we assessed whether HCMV infection will induce a contraction of the HTMCs using a collagen gel contraction assay. To do this, HTMCs were embedded in collagen gel and were assessed for cell contraction after HCMV infection. Our results showed that HCMV infection induced cell contraction significantly more than mock infection (**Figure 7**). Exposure to SB225002 reduced the HCMV-induced cell contraction significantly. SB225002 exposure also reduced cell contraction of uninfected cells. These findings indicated that signaling by CXCR2 is active for infection-induced cell contraction, and it is also required for maintaining homeostasis by maintaining cell tension. In contrast, exposure to RS504393 did not affect cell contraction of the HTMCs.

**Figure 8:**
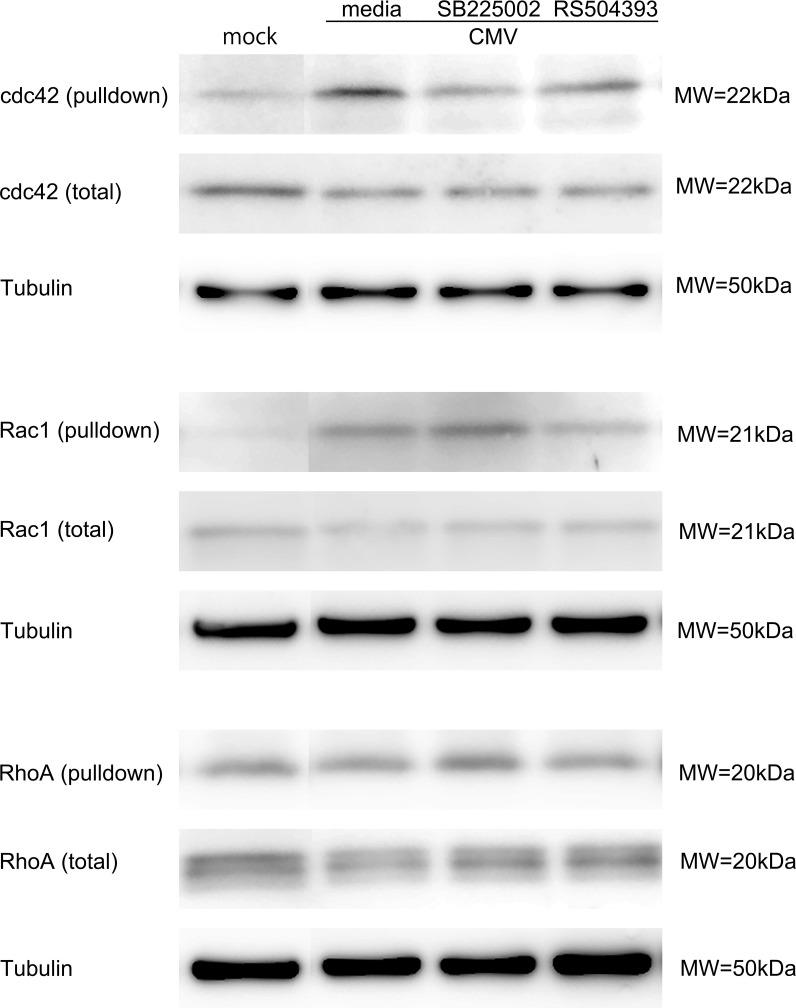
Activation of Rho GTPases after HCMV infection. HTMC cells infected with HCMV at 12 h post infection were assayed by western blot. Rho GTPase activation was determined by pull down assay. For detection of activated RhoA, p21-activated protein kinase bound beads were used. For the detection of activated Cdc42 and Rac1, p21 binding domain of p21-activated protein kinase was used for the pull down. Bands were made visible by anti-RhoA, anti-Cdc42, anti-Rac1 antibodies. HCMV infection activated Cdc42 and Rac1 significantly. CXCR2 blockade by SB225002 (500 nM) reduced the activation of Cdc42. In contrast, CCR2 blockade by SB225002 (500 nM) reduced Rac1 activation. Data represent repeated experiments.

Our network analysis of the CXCR2-dependent genes after HCMV infection indicated that the Rho-kinase pathway is operative for CXCR2-mediated signaling (**Figure 4**). To determine the mechanism controlling the contraction of HTMC cells after infection and to identify possible medication targets, we examined whether low molecular weight G proteins in the Rho family including Rho, Rac, and Cdc42, were activated (**Figure 8**). When infected HTMC cells were assessed for Rho family low molecular weight G protein by activation assays, our results showed that HCMV infection activated Cdc42 and Rac1. This Cdc42 activation was suppressed by blocking CXCR2 (SB225002). In contrast, Rac1 activation was suppressed by blocking CCR2 (RS504393). Thus, CXCR2 and CCR2 play different roles in the activation of Rho GTPases after HCMV infection. Together with the network analyses and cell contraction assays (**Figure 7**), IL-8/CXCR2 using Cdc42 appeared to play crucial roles in HTMCs dynamics after HCMV infection.

## Discussion

Our results have determined the roles played by IL-8 and CCL2 in the pathophysiology of HTMCs following HCMV infections. Our findings showed the involvement of these chemokines in modulating cellular motility and contraction that are key factors in the development of ocular hypertension in virus-associated secondary glaucoma. Understanding these molecular mechanisms is important for advancing our knowledge of the cause of the IOP elevations, and therapeutic approaches for managing IOP elevation in secondary glaucoma.

The TB40/E strain of HCMV used in this study is a well-characterized endothelial-adapted strain that can replicate efficiently in corneal endothelial cells and trabecular meshwork cells (Hosogai et al., 2015; Shimizu et al., 2018). This strain is especially important for studying the viral pathogenesis in conditions such as HCMV anterior uveitis and secondary glaucoma. The clinical relevance of the TB40/E line is its close resemblance to the action of HCMV in human infections, making it a practical model for studying the molecular mechanisms underlying HCMV-induced disorders (Sinzger et al., 2008). (Vo et al., 2020)

Our findings demonstrated that HCMV infection of HTMCs leads to a significant increase in the expression of IL-8 and CCL2 at both the mRNA and protein levels. These findings are consistent with earlier studies that reported elevated levels of IL-8 and CCL2 in the aqueous humor of patients with POAG and hypertensive anterior uveitis (Li et al., 2012; Lee et al., 2021)

In addition to IL-8 and CCL2, a broader spectrum of pro-inflammatory cytokines is elevated in different types of glaucoma. In POAG, IL-6, IL-8, IL-12, IFN-γ, TNF-α, and CXCL9 have been shown to be elevated (Chua et al., 2012; Ghanem et al., 2011; Kuchtey et al., 2010; Sawada et al., 2010). For pseudoexfoliation glaucoma, IL-4, IL-6, IL-8, IL-16, CCL13, CCL15, CCL22, CCL24, CXCL13, and CXCL16 were elevated (Chua et al., 2012; Ghanem et al., 2011; Kuchtey et al., 2010; Sawada et al., 2010)

Earlier, we showed that elevated IOPs were significantly correlated with the intracameral levels of IL-5, IL-6, IL-8, IL-10, IL-15, IL-17, and CCL2 in eyes with pseudoexfoliation glaucoma (Chono et al., 2018). Of these IOP-related cytokines, the elevation of IL-8 was significantly associated with a prognosis for the need of glaucoma surgery (trabeculectomy). For example, IL-8 may stimulate fibrotic remodeling of the outflow system, as was reported for idiopathic pulmonary fibrosis (Yang et al., 2018). CCL2 is generally known to be involved in tissue remodeling, and it stimulates type 1 collagen production in fibroblast (Yamamoto et al., 2000). Thus, the correlation of the elevated IL-8 and CCL2 with IOP can be explained by fibrotic remodeling of the outflow system of the anterior chamber of the eye.

Our analysis of the HTMCs indicated that IL-8 and CCL2 had an immediate effect on cell motility and contraction, which will lead to an increase in the resistance in the outflow system. This contraction was observed after HCMV infection through CXCR2 (**Figure 6**).

Clinically, HCMV induced anterior uveitis or corneal endotheliitis is characterized by very high IOPs, and the IOP is reduced when the level of HCMV is reduced. Thus, viral proliferation appears to directly cause contraction of the HTMCs and elevated resistance of the trabecular system.

Thus, upregulation of these chemokines following viral infection indicates the pathological changes that occur in the HTMCs leads to increased resistance to aqueous humor outflow and subsequent IOP elevation.

In addition, normal TMCs constitutively secrete abundant amounts of IL-8 and CCL2 (Shifera et al., 2010). This suggests homeostatic roles of these chemokines in maintaining cellular tonicity and physiological movements.

Our network analysis of IL-8/CXCR2 responsive genes after HCMV infection showed several key signaling pathways that are essential for maintaining the structure, motility, and function of the TMCs (**Figure 3**). First, the actin cytoskeleton is vital for the translocation of the Solute Carrier Family 2 Member 4, GLUT4 (SLC2A4) to the plasma membrane. This process is essential for the uptake of glucose by cells thereby affecting the homeostasis of cellular energy (Usui et al., 2003).

Second, the integrity of the adherens junctions is important for maintaining the cell-cell adhesions and tissue architecture of the TMCs. Cdc42 and other Rho GTPases regulate the remodeling of these junctions which is necessary for cellular motility.

Third, the Rho GTPases interact with IQGAPs as scaffolding proteins to regulate actin filament organization and cell motility (Brown and Sacks, 2006). For example, activation of IQGAPs by Cdc42 is crucial for the proper formation of filopodia and the maintenance of cellular polarity.

Fourth, aggrephagy, the selective degradation of protein aggregates, is a cellular process that helps maintain protein homeostasis. Aggrephagy is important for preventing the accumulation of damaged proteins (Menzies et al., 2017)

The IL-8-responsive canonical pathway also includes post-translational modifications of tubulin, formin as actin nucleation factor, and kinesins as motor proteins to facilitate vesicle transport (Goode and Eck, 2007; Hirokawa et al., 2009). All of these processes are crucial for maintaining the structural integrity, motility, and cellular organization and function of TMCs that are essential for normal aqueous humor outflow.

Our study highlighted the distinct profiles of the Rho family GTPases-Cdc42, Rho-A, and Rac1 following HCMV infection, especially in the context of IL-8/CXCR2 signaling. Generally, activation of these GTPases plays a pivotal role in regulating the cytoskeletal architecture which directly impacts cell motility and contraction. These cellular changes are crucial for understanding the pathogenesis of virus-induced secondary glaucoma.

We have analyzed the activation profiles of Cdc42, Rho-A, and Rac1 after HCMV infection. Our findings emphasize the unique contributions of the coordinate activation of Cdc42 and Rac1, especially in CXCR2 and CCR2-mediated activation after HCMV infection. Cdc42 is a critical regulator of the actin cytoskeleton and plays a key role in the formation of filopodia which are slender, actin-rich projections that allow cells to explore their environment and interact with the extracellular matrix (Heasman and Ridley, 2008). Rac1 activation is known to be involved in the formation of lamellipodia, which are broad, flat protrusions which enable cell spreading and movement (Steffen et al., 2013). In contrast, Rho-A primarily regulates the formation of the stress fibers. During stress fiber formation in TMCs, RhoA undergoes post-translational modifications, leading to a shift in its molecular weight.(**Figure 8**) (Stubbs and Von Zee, 2012) The activation of Cdc42 by the IL-8/CXCR2 axis in TMCs after HCMV infection suggests that Cdc42 is especially important for initiating the cytoskeletal changes which may lead to the increased outflow resistance associated with viral infections.

The differential activation of these GTPases underscores the complexity of the regulation of TMCs and suggests potential therapeutic targets for managing elevated IOP in secondary glaucoma. Specifically, targeting the IL-8/CXCR2/Cdc42 axis could provide a new approach to modulate TM cell motility and contraction leading to reducing outflow resistance and preventing the progression of glaucoma. Understanding these molecular pathways in greater detail will be critical for developing targeted therapies that can effectively address the cytoskeletal dysregulation observed in TM cells following viral infection.

Our study has some limitations. Our observations were based on cultured TMCs and may not mirror the physiological conditions observed in HCMV infected individuals. In addition, cellular contraction and movement are affected by the extracellular matrix which may be different in *in vivo* conditions. However, our study provides important information on how TMCs behave after HCMV infection and the possible roles of IL-8 and CCL2.

## Conclusion

Our results determined the distinct roles of the Rho GTPases regulating the cytoskeletal dynamics of TMCs following HCMV infection. The specific activation of Cdc42 by the IL-8/CXCR2 pathway emphasizes its critical role in the pathophysiology of viral-induced glaucoma, offering potential avenues for therapeutic intervention.

## Materials and methods

### Cells and virus

Human trabecular meshwork cells (HTMCs) were isolated from the juxtacanalicular and corneoscleral regions of cadaver eyes donated for research purposes (#6590, ScienCell, Carlsbad, CA). The HTMCs were propagated to confluence on fibronectin coated 6- or 96-well plates in trabecular meshwork cell media supplemented with 2% fetal bovine serum (ScienCell).

The TB40/E strain of HCMV was propagated on human foreskin fibroblast cells (HFF) that were transfected by a TB40-BAC4 clone (Shimizu et al., 2018). The viral titers were measured using the 50% tissue culture infection dose (TCID50) method. HTMC cells were stimulated with 10 ng/ml of IL-8 (Peprotech, Cranbury, NJ) or 10 ng/ml CCL2 (Peprotech). For HCMV infection, HTMC were infected at multiplicity of infection (MOI) 1. For blocking experiments, HTMC were infected by TB40/E at MOI 1 and exposed to CXCR2 or CCR2 antagonists. For blocking CXCR2, an IL-8 receptor, 500 nM of SB225002 (CXCR2 antagonist, Abcam, Cambridge, UK) was used. For blocking CCR2, 5 µM RS504393 (CCR2 antagonist, Tocris, Bristol, UK) was used.

### Real time RT-PCR

Total RNA was isolated from HTMCs (2×10^5^ cells/well) after infection at multiplicity of infection (MOI) 1 using the RNeasy mini kit (Qiagen), and reverse transcribed using the QuantiTect Reverse Transcription Kit (Qiagen). The cDNAs were amplified using the QuantiTect SYBR Green PCR kit and normalized to the 18S rRNA using Ct values. The sequences of the real-time PCR primer pairs were shown in **Table 1**.

**Table 1:** Primers for real-time PCR.

IL-8	Forward 5′- CTTGGCAGCCTTCCTGATTT-3′,
	Reverse 5′- TTCTTTAGCACTCCTTGGCAAAA-3′
CCL2	Forward 5′-AGGTGACTGGGGCATTGAT-3′,
	Reverse 5′-GCCTCCAGCATGAAAGTCTC-3′
18S rRNA	Forward 5’-GGCCCTGTAATTGGAATGAGTC-3’
	Reverse 5’-CCAAGATCCAACTACGAGCTT-3’

### RNA-seq analysis

HTMC infected by TB40/E (2×10^5^ cells/well, N=4/group) were exposed to CXCR2 antagonist (500 nM of SB225002) or CCR2 antagonist (5 µM of RS504393). Total RNA was isolated from the HTMCs after 12 h post infection (PI) using the RNeasy mini kit (Qiagen). An RNA library was prepared using the Ion AmpliSeq Transcriptome Human Gene Expression Kit (Thermo Fisher Scientific, Waltham, MA), and sequenced using the Ion Proton System (Thermo Fisher Scientific).

The mapped sequencing read data as BAM files were analyzed using featureCounts. Differential expression was analyzed using edgeR. A false discovery rate (FDR) <0.05 was considered significant. (DOI: 10.5281/zenodo.14543758) (https://zenodo.org/records/14543758)

### ELISA

Supernatants of HTMC cells after HCMV infection (2×10^5^ cells/well) were assayed for the levels of IL-8 and CCL2 using commercially available ELISA kits (IL-8, BioLegend, San Diego, CA; CCL2, PeproTech). The concentrations of IL-8 and CCL2 were determined according to the manufacturer’s instructions.

### Western blot

Cell lysates (6×10^5^ cells/well) of TB40/E infected HTMC at MOI 1 were harvested at 12 h post infection, and 10 μg of the protein/lane were electrophoresed. The transferred membranes were stained with antibodies for Rac1, Cdc42, RhoA, and tubulin. Rho GTPase activation assays were conducted using Rho Activation Assay Biochem Kit (Cytoskeleton, Denver, CO) and Rac1/Cdc42 Activation Magnetic Beads Pulldown Assay (Merck, Darmstadt, Germany). Briefly, the active form of RhoA was pulled down by Rhotekin-Rho binding domain conjugated beads. The bound active form of GTP-Rho was detected by immunoblotting using mouse anti-human monoclonal IgM RhoA antibody (Cytoskeleton).

To detect the active form of Rac1 and Cdc42, p21-activated protein kinase (PAK1), which is a downstream effector of Rac/Cdc42, was used. The p21 binding domain (PBD) of PAK1 conjugated to magnetic beads was used to pull down the active form of Rac/Cdc42. The bound active forms of Rac/Cdc42 were detected by immunoblotting using mouse anti-Rac1 monoclonal antibody (clone 23A8) or mouse monoclonal anti-Cdc42 antibody. The immunoreactive bands were made visible by goat anti-mouse horseradish peroxidase-conjugated secondary antibody and enhanced chemiluminescence.

### Assessment of cell motility

The HTMCs were plated on fibronectin-coated 96-well plates at 10^3^ cells/well in TMC media supplemented with 2% fetal bovine serum. Plated HTMCs were infected with HCMV at MOI of 1 and stained for actin with SiR-actin (Spirochrome, Stein am Rhein, Switzerland) according to the manufacturer’s instructions.

Actin-stained HTMC cells were made visible for examination by Fluorescence microscopy with 652 nm excitation (BZ-X810, Keyence, Osaka Japan). Time-lapse images of the live cells were continuously photographed under 5% CO_2_ at 37°C up to 24 hours after exposure and analyzed using time-lapse module (BZ-H4XT, Keyence).

For analysis, 4 three-dimensional frames of 1410 μm × 1060 μm × 90 μm were analyzed. Cell tracking and measurement of cell movement was analyzed using BZ-X800 Analyzer software (Keyence).

### Collagen gel contraction assay

Confluently grown HTMCs were infected with HCMV at MOI 1 for 1 hour. Infected or mock infected HTMC cells (5×10^4^ cells/well) were resuspended and mixed with type I human collagen solution (VitroCol, Advanced Biomatrix, Carlsbad, CA) according to the manufacturer’s instructions (Luna et al., 2012). The cell embedded collagen was polymerized at room temperature for 30 minutes on 24 well plates. After collagen polymerization, media containing SB225002 or RS504393 were supplemented. Collagen gels were detached from the walls, and the gel area was quantified after 6 h using Photoshop (Adobe, San Jose, CA). Gel contraction was calculated using the formula: Gel Contraction = 100 – percentage of Gel Area

### Statistical analyses

Data are presented as the means ± standard error of the means (SEMs). The significance of differences was determined by *t* tests, Mann Whitney U tests, ANOVA with *post hoc* tests, or multivariate linear regression analysis.

All protocols and methods adhered to the tenets of the Declaration of Helsinki.

## Data Availability

The datasets presented in this study can be found in online repositories. The names of the repository/repositories and accession number(s) can be found below: https://zenodo.org/records/14543758, DOI: 10.5281/zenodo.14543758.

## Supplementary material

Supplementary Video HCMV induced cell motility of human trabecular meshwork cells (HTMCs). HTMCs infected with TB40/E were stained for actin using SiR-actin. HCMV infection increased the cell motility. Increased cell motility was accompanied by formation of lamellipodia and filopodia. Lamellipodia is a broad, flat protrusion that aids in cell migration by enabling cell spreading and movement. Filopodia are thin actin rich protrusions from the cell surface that are crucial in sensing cellular environment and initiating cell migration. IL-8 or CCL2 treatment also promoted cell migration. IL-8 treatment was associated with formation of both lamellipodia and filopodia, whereas CCL2 treatment was mainly characterized by lamellipodia.
