# Supplementary figures and images for: Roles played by IL-8 in altering dynamics of trabecular meshwork cells after human cytomegalovirus infection
Source: Front Cell Infect Microbiol. 2025 Apr 25;15:1550509. doi: 10.3389/fcimb.2025.1550509 (PMC12061996; doi:10.3389/fcimb.2025.1550509)

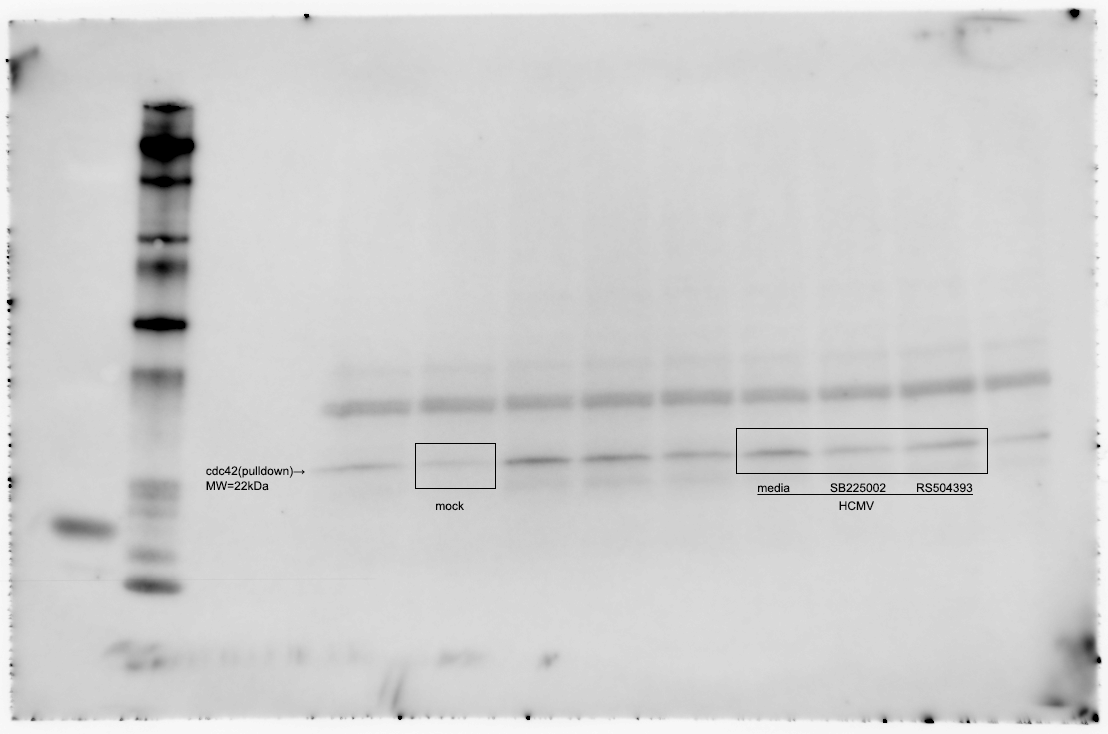

Supplement: Supplementary file 5 [file Image1.tif]

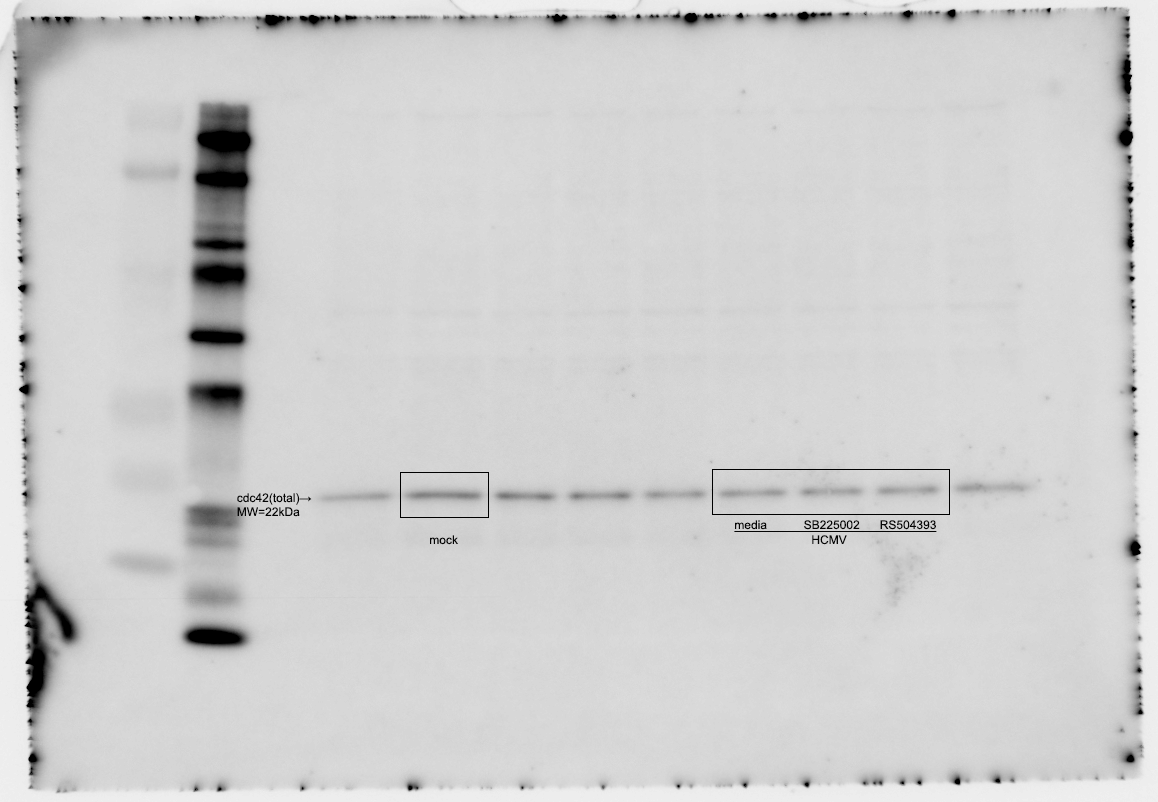

Supplement: Supplementary file 6 [file Image2.tif]

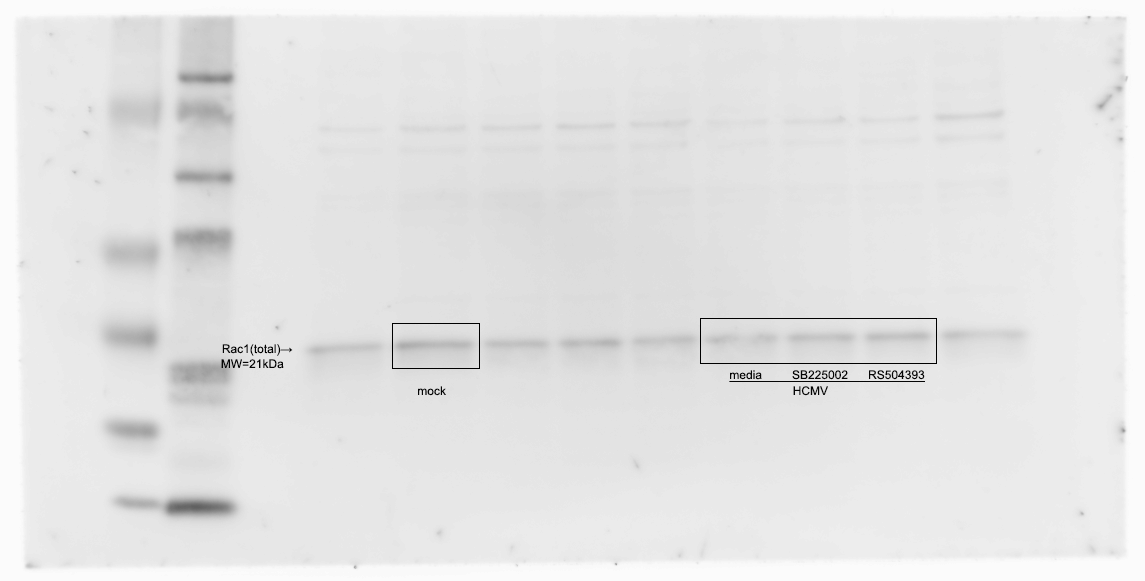

Supplement: Supplementary file 7 [file Image3.tif]

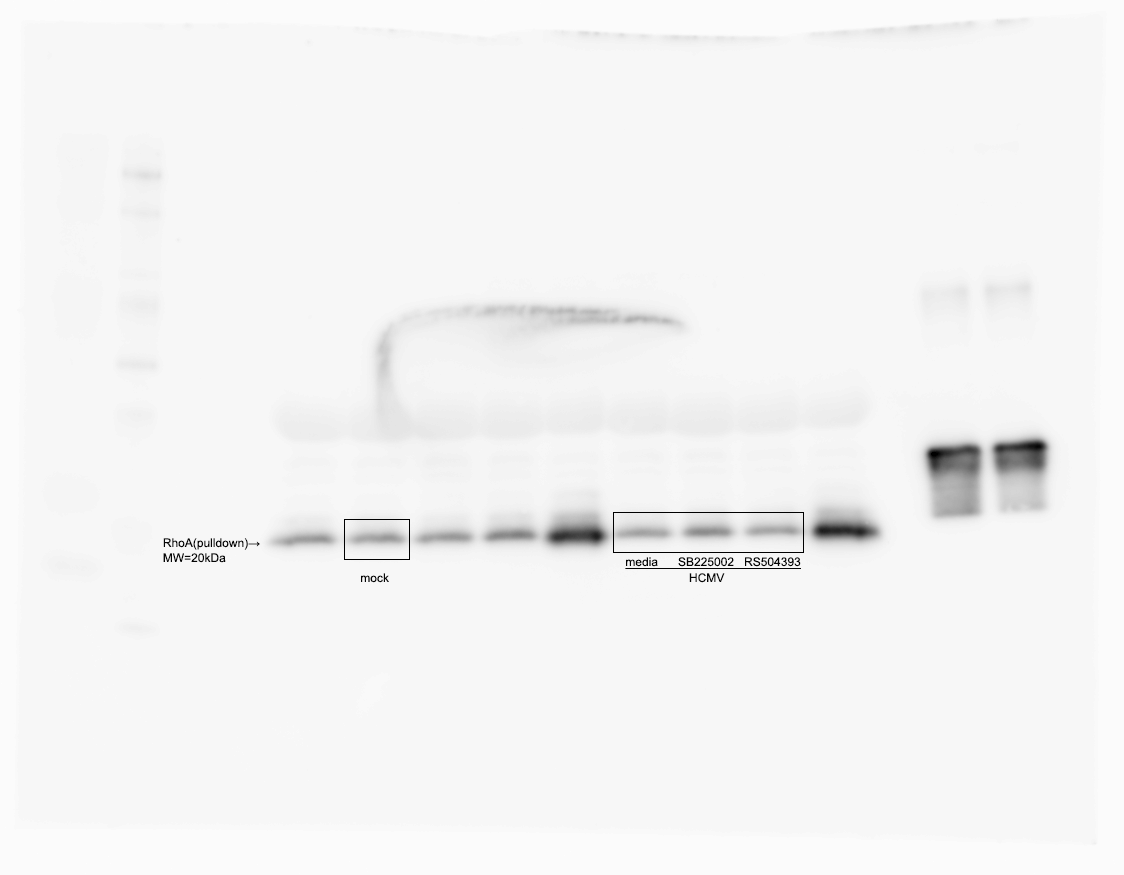

Supplement: Supplementary file 8 [file Image4.tif]

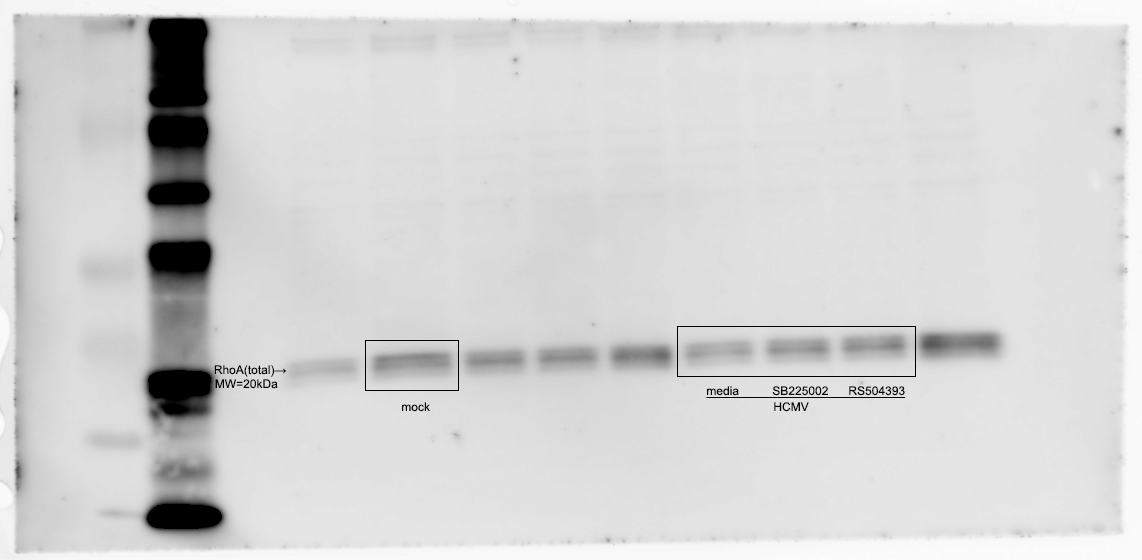

Supplement: Supplementary file 9 [file Image5.tif]

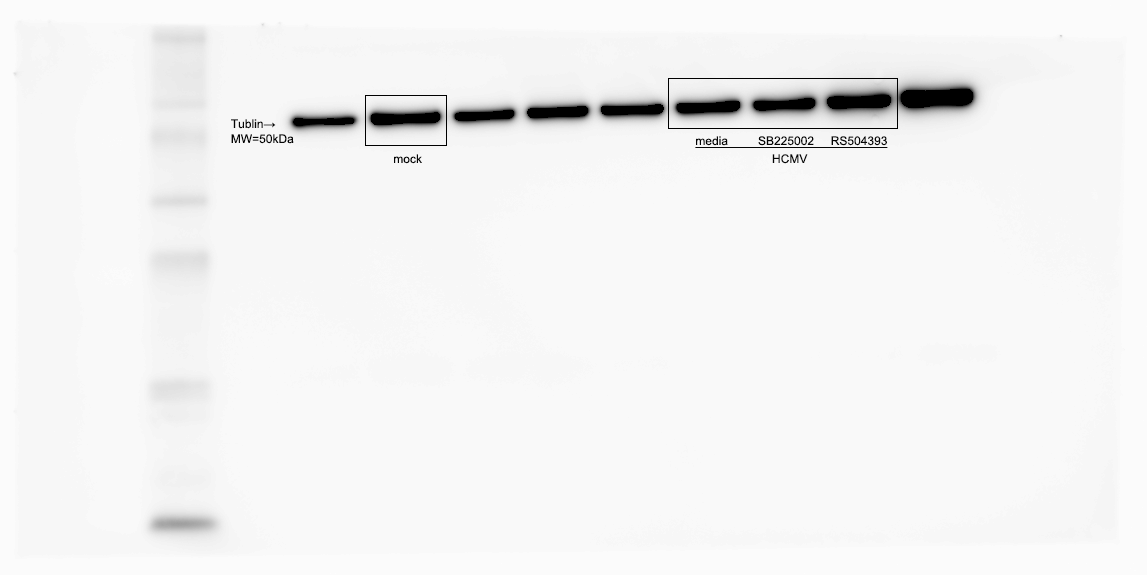

Supplement: Supplementary file 10 [file Image6.tif]

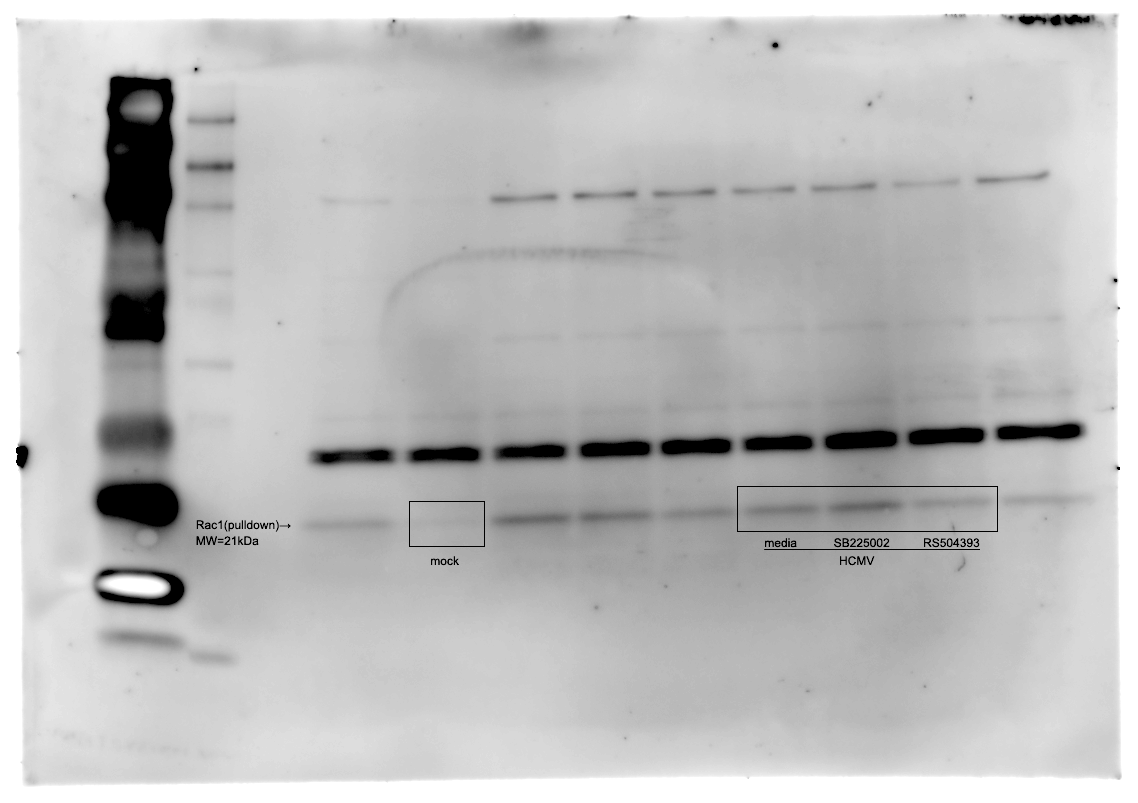

Supplement: Supplementary file 11 [file Image7.tif]
